# The role of spleen in the treatment of experimental lipopolysaccharide-induced sepsis with dexmedetomidine

**DOI:** 10.1186/s40064-015-1598-y

**Published:** 2015-12-22

**Authors:** Zhaoguo Liu, Yaoqi Wang, Qiaoqing Ning, Chunzhi Gong, Yong Zhang, Li Zhang, Xiangmei Bu, Guangjian Jing

**Affiliations:** Department of Anesthesiology, Binzhou Medical University Hospital, Binzhou, 256603 Shandong People’s Republic of China

**Keywords:** Spleen, Dexmedetomidine, Cholinergic anti-inflammatory pathway, Sepsis, Lipopolysaccharides

## Abstract

Dexmedetomidine (Dex), a highly selective α2-adrenergic receptor agonist, has been shown to attenuate systemic inflammatory response induced by lipopolysaccharide (LPS). The protective effects of Dex may reportedly be due to the activation of the α7 nicotinic acetylcholine receptor (α7nAChR)-dependent cholinergic anti-inflammatory pathway. Spleen has been shown to play a pivotal role in the neural cholinergic anti-inflammatory pathway. However, little is known about the specific function of spleen in the protective effects of Dex against sepsis. To investigate the role of spleen in the treatment of Dex against sepsis, we studied the effects of preemptive administration of Dex to septic mice on the NF-κB p65 activation and downstream pro-inflammatory cytokine expression in the spleen. Our results provided evidence that Dex treatment attenuated LPS-activated NF-κB p65 activation, as well as the production of tumor necrosis factor-α, interleukin-6, and interleukin-1β at the level of both mRNA and protein in spleen. Consequently, serum concentrations of these cytokines decreased. Conversely, preemptive injection of α-bungarotoxin, a selective α7nAChR antagonist, reversed these effects of Dex. Our findings indicated that spleen played a critical role in the protective effects of Dex against sepsis and provided further insight into the anti-inflammatory mechanisms of Dex.

## Background

Sepsis is a condition characterized by uncontrolled infection and affects many organs, with an attendant high mortality rate (Angus et al. 2001). The onset and perpetuation of sepsis involve the up-regulation of NF-κB (Müller et al. 1993) and a complex network of cytokines, such as tumor necrosis factor-α (TNF-α), interleukin-6 (IL-6), and interleukin-1β (IL-1β) (Beutler and Cerami 1989; Durum et al. 1985; Hofer et al. 2009; Wong and Clark 1988). The unbalanced overproduction of these mediators provokes overwhelming inflammation responses and eventual lethal multiple-organ failure. Therefore, reducing excessive inflammation cytokine production during sepsis is beneficial to relieve the excessive inflammatory responses of septic mice.

Dexmedetomidine (Dex), a highly selective α2-adrenergic receptor agonist, is widely used for sedation in intensive care units and in clinical anesthesia (Carollo et al. 2008). Dex also has good perioperative hemodynamic stability and reduces intraoperative anesthetic requirements (Arcangeli et al. 2009). Earlier research has shown that Dex can regulate systemic inflammatory response by decreasing the synthesis of proinflammation cytokines, such as TNF-α, IL-6, and IL-1β (Hofer et al. 2009; Taniguchi et al. 2008). Other studies have shown that the anti-inflammatory mechanism of Dex may be associated with a reduction in the central sympathetic tone by stimulating central α2 receptors in the medulla oblongata (Pandharipande et al. 2007; Rittirsch et al. 2008), resulting in the balance shifts to the benefit of the parasympathetic nervous system. This phenomenon activates the efferent vagus nerve, leading to the suppression of biosynthesis and release of pro-inflammatory cytokines. This efferent neural signaling pathway is termed the cholinergic anti-inflammatory pathway (Borovikova et al. 2000), a neurophysiological mechanism that regulates the immune system via the release of acetylcholine, which can interact with α7nAchR expressed by macrophages and other cytokine-producing cells, resulting in the inhibition of NF-κB activation and proinflammatory cytokines synthesis, thereby preventing tissue damage (Parrish et al. 2008; Saeed et al. 2005; Tracey 2007). A previous investigation has shown that the effects of vagal stimulation on serum TNF, IL-6, and IL-1 β production were lost in α7nAChR knockout animals, confirming that the receptor is the key in the vagal mechanisms of immunosuppression (Wang et al. 2003).

Extensive earlier investigations have reported that spleen, the main source of circulating pro-inflammatory cytokines, is the target organ of the vagus nerve for controlling pro-inflammatory cytokine production (Huston et al. 2006; Rosas-Ballina et al. 2008). An anatomical study has shown that the common coeliac branch of the vagus nerve carries the parasympathetic fibers to the splenic ganglion. Selective nerve interruption further confirms it to be the branch responsible for the efferent arm of the anti-inflammatory pathway to the spleen (Huston et al. 2006). Thus, we hypothesized that Dex may activate the cholinergic anti-inflammatory pathway, thereby inhibiting inflammatory cytokine synthesis in spleen, resulting in reduced overwhelming inflammatory responses to sepsis. However, few studies have proven this hypothesis. In this study, we used a mice model of lipopolysaccharide (LPS)-activated sepsis, mimicking what occurs in clinical sepsis, to investigate the effects of Dex and α-Bgt on spleen TNF-α, IL-6, and IL-1β release in vivo. To the best of our knowledge, this study is the first one that may confirm the role of spleen in the protective effects of Dex against septic mice.

## Methods

### Animals

Male BALB/c mice weighing 18–20 g were obtained from Chang Zhou Cavens Laboratory Animal Ltd. (Changzhou, China). The animals were kept at 23 °C with 12 h light/12 h dark cycles each day and allowed free access to water and food. The investigation was in compliance with the Guide for the Care and Use of Laboratory Animals published by the National Institutes of Health, and all experiments were approved by the Institutional Animal Care and Use Committee of Binzhou Medical University (Yantai, China).

### Chemicals

Dex was obtained from Jiangsu Hengrui Medicine Co., Ltd. (Jiangsu, China). Mouse TNF-α, IL-6, and IL-1β assay kits were purchased from Shanghai Lengton Bioscience Co., Ltd. (Shanghai, China). LPS (*Escherichia coli* 0111: B4) and α-Bgt were purchased from Sigma Chemical Co. (St. Louis, MO, USA). Nuclear and cytoplasmic protein extraction reagent kits were obtained from Beyotime Institute of Biotechnology (Shanghai, China). Anti-TNF-α, anti-IL-6, anti-IL-1β, anti-p-IκB p65, anti-IκB p65, anti-NF-κB p65, anti-Lamin B, and anti-β-actin monoclonal antibodies were purchased from Cell Signaling Technology (Danvers, MA, USA). All other chemicals were reagent grade.

### Experimental design

In the other experiments, mice were randomly divided into five groups (n = 16 per group): control, LPS, Dex + LPS, α-Bgt + Dex + LPS, α-Bgt + LPS. Dex (40 μg/kg) was administered intraperitoneally 15 min prior to LPS (10 mg/kg) challenge, α-Bgt (1 μg/kg) was added 15 min before LPS or Dex, and control group mice received only normal saline. The chosen doses of these drugs were based on our preliminary experiments and previous studies (Xiang et al. 2014). About 2 h after saline/LPS administration, the 16 mice of each group were randomly divided equally into two, and all mice were sacrificed with an overdose of pentobarbital (60–90 mg/kg) injected intraperitoneally. Serum samples were collected immediately. Part 1 was used for spleen inflammatory cytokine determination and immunohistochemical examination. The superior part of the spleen tissues was harvested, placed in appropriate amount of precooled PBS, and homogenized immediately. Then, the homogenate was centrifuged at 4 °C, and the supernatant was collected for further analysis of TNF-α, IL-6, and IL-1β. At the same time, the lower part of spleen tissues was harvested for immunohistochemical examination. Part 2 was left for real-time polymerase chain reaction (RT-PCR) assay and Western blotting. The superior part of the spleen tissues was used to extract total RNA for RT-PCR measurement, and the lower part of the spleen tissues were used to extract nuclear and cytoplasmic proteins for Western blotting.

### ELISA assay for TNF-α, IL-6, and IL-1β in serum

Serum samples were collected for TNF-α, IL-6, and IL-1β detection. According to the manufacturer’s instructions, the concentrations of all cytokine (TNF-α, IL-6, and IL-1β) were detected by ELISA kits. A standard curve was constructed using various dilutions of TNF-α, IL-6, and IL-1β standard preparation. The levels of these cytokines were calculated according to standard curves.

### ELISA assay for TNF-α, IL-6, and IL-1β in spleen homogenates

Mice spleen tissues were placed in an appropriate amount of precooled PBS and then homogenized to determine the levels of inflammation cytokines. TNF-α, IL-6, and IL-1β in spleen homogenates were measured using commercially available ELISA kits. The levels of TNF-α, IL-6, and IL-1β were calculated according to standard curves.

### RT-PCR assay for TNF-α, IL-6, and IL-1β mRNA expression in spleen tissues

The levels of TNF-α, IL-6, and IL-1β mRNA in spleen tissues were detected by RT-PCR. Total RNA from spleen tissues was extracted using Trizol reagent. First-strand cDNA was synthesized following the manufacturer’s instructions for a Transcriptor One Step RT-PCR Kit (Roche, Switzerland). The primers were as follows: TNF-α, forward CACCACCATCAAGGACTCAA, reverse GAGACAGAGGCAACCTGACC; IL-6, forward CGGAGAGGAGACTTCACAGAG, reverse CATTTCCACGATTTCCCAGA; IL-1β, forward CTCACAAGCAGAGCACAAGC, reverse TCCAGCCCATACTTTAGGAAGA; β-actin, forward GTGCTATGTTG CTCTAGACTTCG, reverse ATGCCACAGGATTCCATACC. We calculated the mean fold change in the expression of TNF-α, IL-6, and IL-1β mRNA in the experimental group compared with the control group. β-Actin was used as the reference gene. Results were expressed as relative fold compared with control animals.

### Immunohistochemical assay for NF-κB p65 activation

NF-κB p65 protein activation was observed by immunohistochemistry technique. In a typical procedure, sections were microwaved for antigen retrieval and pretreated with 0.3 % H_2_O_2_. Subsequently, the sections were blocked with goat serum and incubated in a primary antibody solution containing rabbit anti-NF-κB p65 antibody overnight at 4 °C. After washing, the samples were incubated in a suitable secondary antibody solution for 1 h at room temperature. Finally, the sections were incubated in HRP—streptavidin for 1 h at room temperature, and the color reaction was developed with diaminobenzidine. The sections were counterstained, dehydrated, and analyzed under a light microscope. The degree of NF-κB activation was expressed as percentage of nuclear NF-κB p65 positive cells to total alveolar epithelial cells.

### Western blotting for TNF-α, IL-6, IL-1β,NF-κB p65, p-IκB, and IκB

The levels of TNF-α, IL-6, IL-1β, NF-κB p65, p-IκB, and IκB protein in spleen tissues were assayed by Western blotting. The proteins were separated on 10 % SDS—polyacrylamide gels and transferred onto a PVDF membrane. After blocking the nonspecific site, the membrane was incubated overnight with specific primary antibody at 4 °C. Subsequently, the membrane was washed using TBS with Tween (TBST) and then incubated with the secondary antibody conjugated with horseradish peroxidase at room temperature for 1 h. Blots were again washed with TBST and then developed with the ECL Plus Western Blotting Detection System (Amersham Life Science, UK).

## Results

### Effects of Dex and α-Bgt on serum TNF-α, IL-6, and IL-1β

**Fig. 1:**
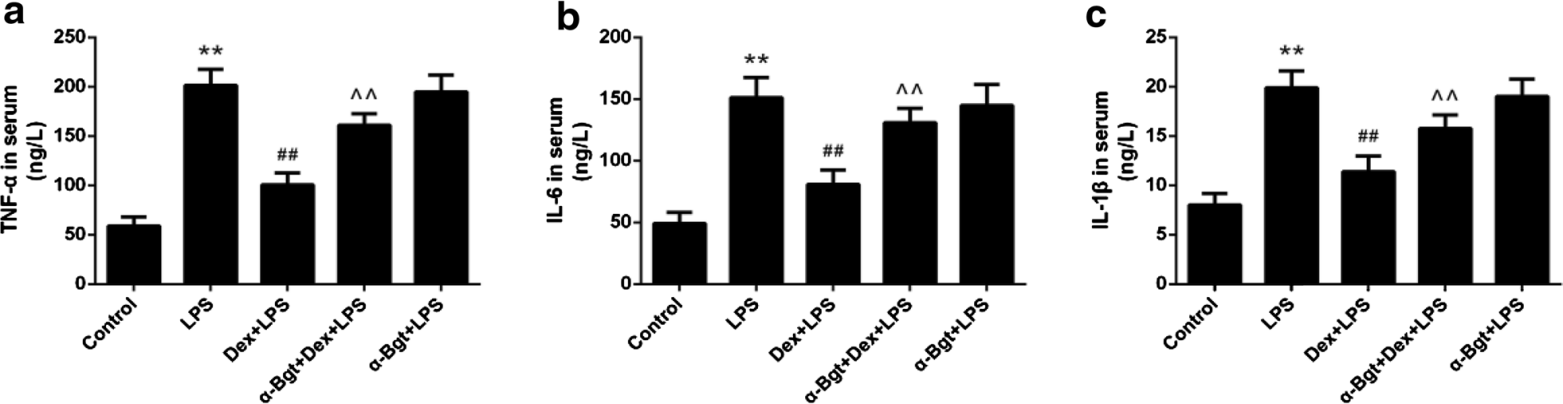
ELISA assay for serum TNF-α (**a**), IL-6 (**b**) and IL-1β (**c**). Data represented the mean ± SEM (n = 8). **p < 0.01 compared to control group; ^##^p < 0.01 compared to LPS group; ^^p < 0.01 compared to Dex + LPS group

As illustrated in Fig. 1, LPS administration obviously enhanced serum concentrations of TNF-α, IL-6, and IL-1β, whereas pretreatment with Dex obviously reduced these cytokines’ contents in serum. Furthermore, the decrease induced by Dex could be substantially reversed by preemptive administration with α-Bgt. No significant differences were found between α-Bgt + LPS and LPS groups in terms of the serum levels of TNF-α, IL-6, and IL-1β.

### Effects of Dex and α-Bgt on TNF-α, IL-6, and IL-1β levels in spleen tissues

**Fig. 2:**
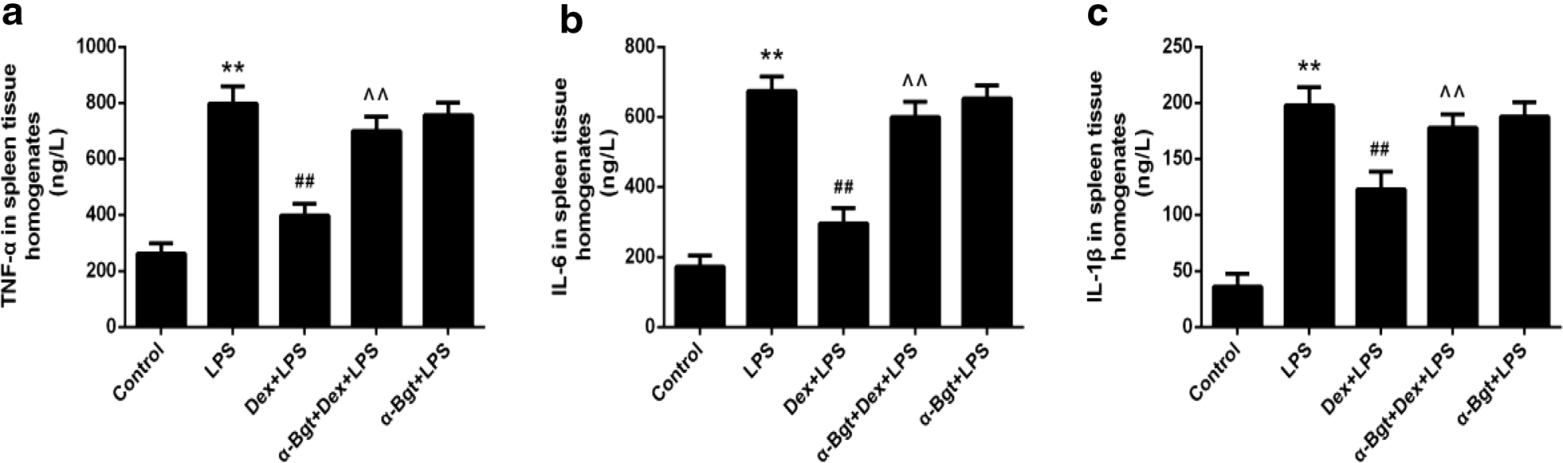
ELISA assay for TNF-α (**a**), IL-6 (**b**) and IL-1β (**c**) concentrations in spleen homogenates. Data represented the mean ± SEM (n = 8). **p < 0.01 compared to control group; ^##^p < 0.01 compared to LPS group; ^^p < 0.01 compared to Dex + LPS group

**Fig. 3:**
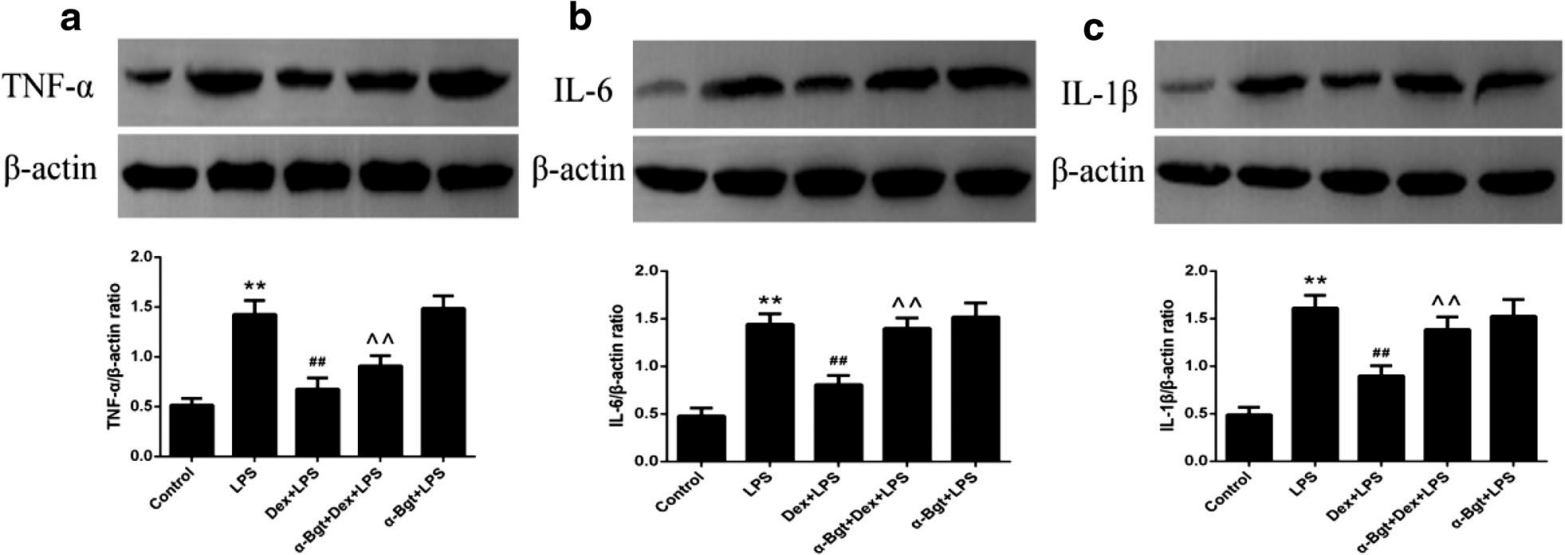
Western blotting assay for TNF-α (**a**), IL-6 (**b**) and IL-1β (**c**) levels in spleen tissues. Data represented the mean ± SEM (n = 8). **p < 0.01 compared to control group; ^##^p < 0.01 compared to LPS group; ^^p < 0.01 compared to Dex + LPS group

As shown in Figs. 2 and 3, we found that pretreatment with Dex efficiently suppressed the LPS-induced increase in TNF-α, IL-6, and IL-1β in spleen tissues. However, the protective effects were obviously reversed by α-Bgt, resulting in elevated TNF-α, IL-6, and IL-1β production. No statistical differences existed between the α-Bgt + LPS and LPS groups for TNF-α, IL-6, and IL-1β expression in spleen.

### Effects of Dex and α-Bgt on TNF-α, IL-6, and IL-1β mRNA expression in spleen tissues

**Fig. 4:**
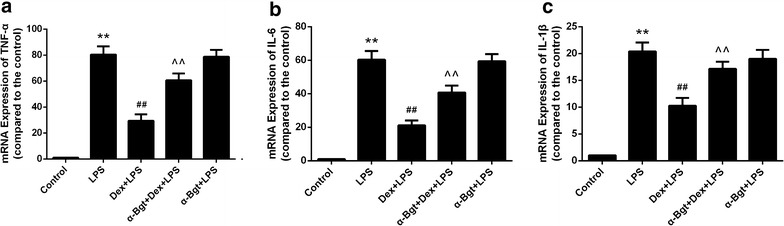
RT-PCR assay for TNF-α (**a**), IL-6 (**b**) and IL-1β (**c**) mRNA in spleen tissues. The levels of Data represented the mean ± SEM (n = 8). **p < 0.01 compared to control group; ^##^p < 0.01 compared to LPS group; ^^p < 0.01 compared to Dex + LPS group

To gain further insight into the anti-inflammatory mechanism of Dex, we determined the mRNA levels of TNF-α, IL-6, and IL-1β. As shown in Fig. 4, Dex attenuated the LPS-activated increase in TNF-α, IL-6, and IL-1β mRNA secretion. Moreover, the inhibitory effects of Dex were abated by α-Bgt.

### Effects of Dex and α-Bgt on LPS-induced NF-κB p65 activation

**Fig. 5:**
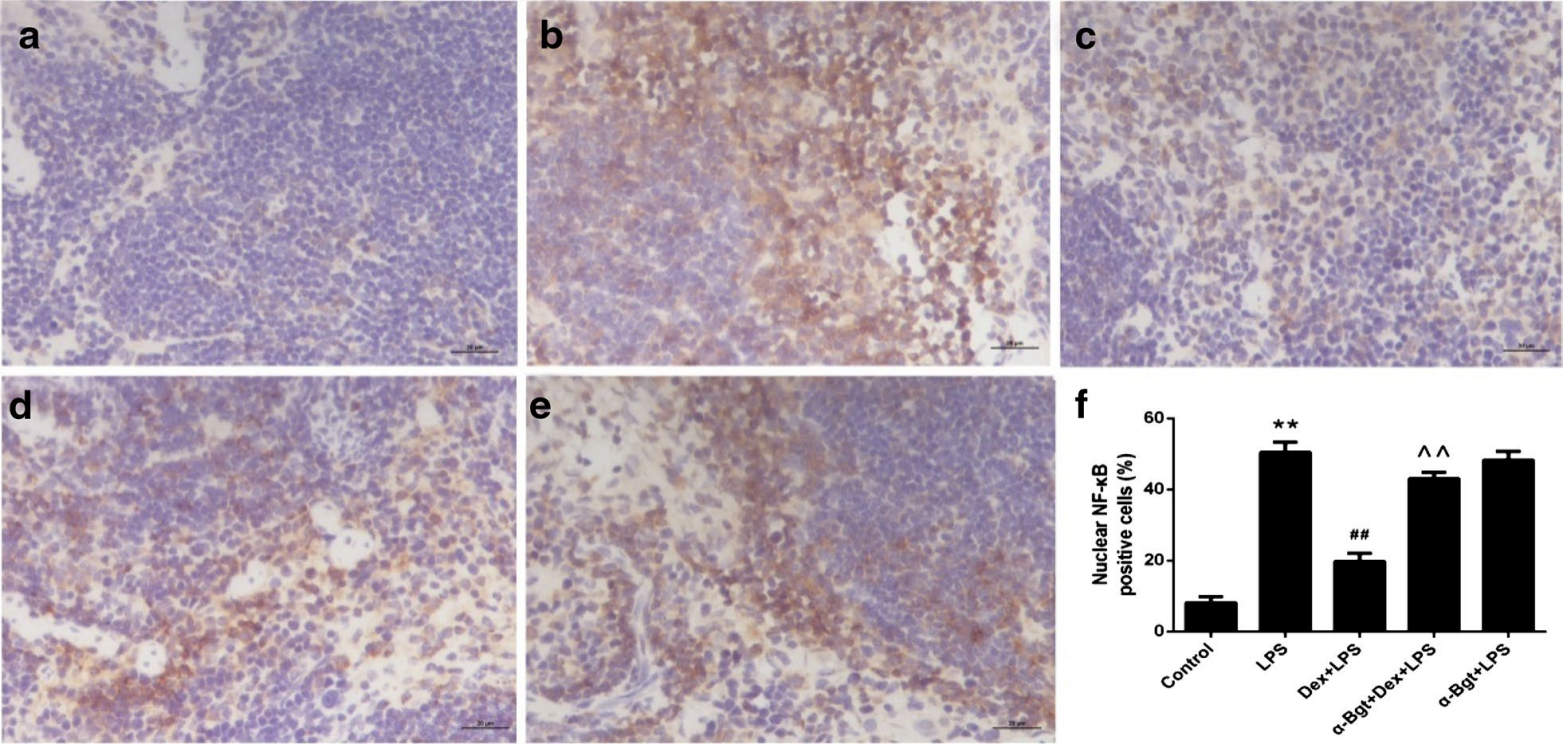
Immunohistochemical assay for NF-κB p65 activation in spleen tissues. Control group (**a**); LPS group (**b**); Dex + LPS group (**c**); α-Bgt + Dex + LPS group (**d**); α-Bgt + LPS group (**e**): magnification ×400. The percentage of nuclear NF-κB positive cells in spleen (**f**). Data represented the mean ± SEM (n = 8). **p < 0.01 compared to control group; ^##^p < 0.01 compared to LPS group; ^^p < 0.01 compared to Dex + LPS group

Based on the above conclusions, we next explored whether the upstream NF-κB signal transduction pathway was also involved. Nuclear positive staining represents the activated form of NF-κB. As shown in Fig. 5, our results indicated that nuclear positive staining was elevated in septic mouse stimulated with LPS, whereas preemptive injection of Dex significantly reduced nuclear positive staining. Moreover, the inhibitory effects of Dex could be significantly abrogated by α-Bgt, leading to an increase in the percentage of cells showing nuclear positive staining. No statistical differences were found between α-Bgt + LPS and LPS groups in terms of nuclear positive staining in spleen tissues.

**Fig. 6:**
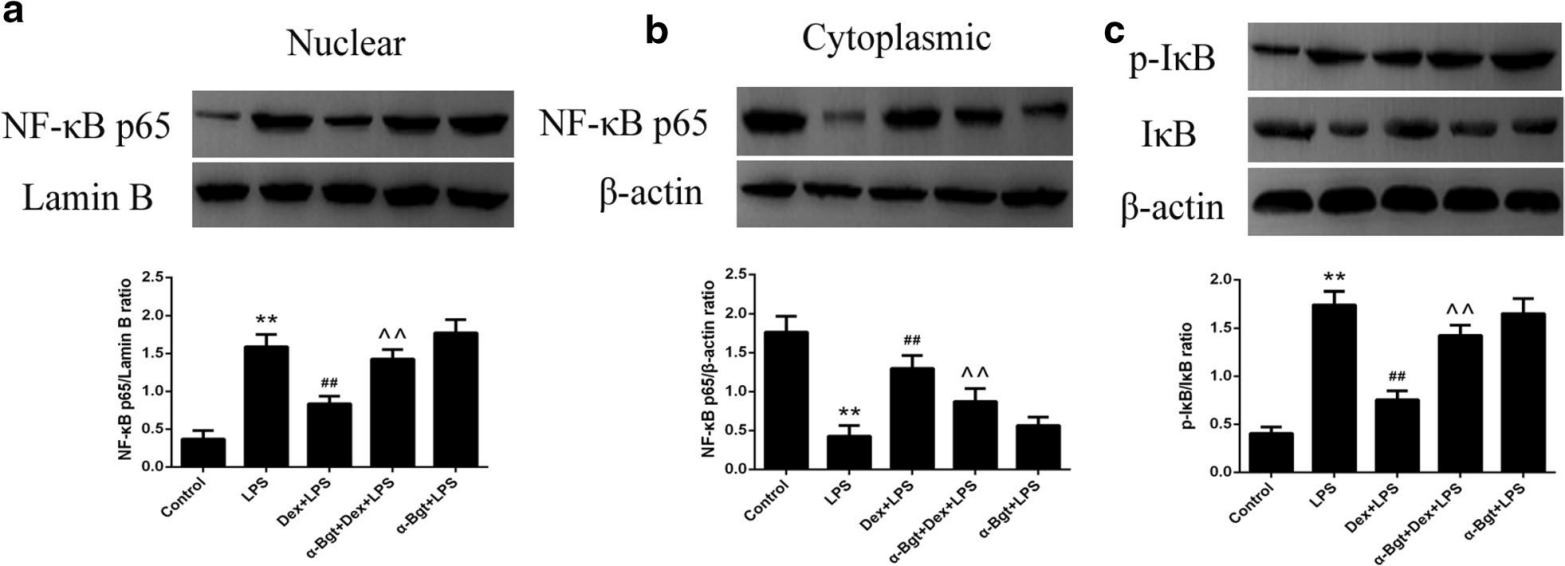
Western blotting assay for NF-κB p65 nuclear translocation and IκB phosphorylation in spleen tissues. The levels of NF-κB p65 in nucleus (**a**), NF-κB p65 in cytoplasm (**b**) and p-IκB/IκB in cytoplasm (**c**). Data represented the mean ± SEM (n = 8). **p < 0.01 compared to control group; ^##^p < 0.01 compared to LPS group; ^^p < 0.01 compared to Dex + LPS group

### Effects of Dex and α-Bgt on LPS-induced IκB phosphorylation and NF-κB p65 activation

As previous studies have shown, activated NF-κB should translocate to the nucleus and bind to the promoter region of multiple genes, including cytokine genes, thereby inducing the expression of cytokine mRNA and protein [19,20]. As shown in Fig. 6, the translocation of NF-κB p65 to nucleus and IκB phosphorylation in spleen significantly increased 2 h after LPS challenge. Moreover, Dex administration prior to LPS decreased NF-κB p65 activation and IκB phosphorylation compared with the LPS group. However, all these decreases were blocked by α-Bgt pretreatment in the α-Bgt + Dex + LPS group. No statistical differences were observed between α-Bgt + LPS and LPS groups in terms of NF-κB p65 activity and IκB phosphorylation.

## Discussion

During sepsis, pro-inflammatory cytokines are known to be released largely unopposed, which leads to pathological injuries (Raetz and Whitfield 2002). In the present study, data showed that serum TNF-α, IL-6, and IL-1β increased following LPS administration, and preventive treatment with Dex relieved the stimulated rise in circulating levels of these pro-inflammatory cytokines. The obvious and rapid decline in the levels of these mediators may contribute to a reduction in multiple organ dysfunctions, resulting in an improvement of outcome in septic mice. Moreover, preventive injection of α-Bgt reversed the Dex-induced reduced severity of experimental sepsis, consistent with several original studies that have also shown Dex possessing anti-inflammatory capacity by activating the cholinergic anti-inflammatory pathway (Xiang et al. 2014).

Removing the spleen itself also reportedly lowers the production and plasma levels of inflammatory cytokines to the same degree as does vagal stimulation when spleen is intact. Thus, spleen is essential for inflammatory regulation in the cholinergic anti-inflammatory pathway (Ramsay and Luterman 2004). However, no studies have been conducted so far to study the role of spleen in the anti-inflammatory effects of Dex. To investigate the specific functions of spleen in the protective effects of Dex, we analyzed the changes in pro-inflammatory cytokine levels in spleen. Dex stimuli was found to suppress the production of TNF-α, IL-6, and IL-1β in spleen, and α-Bgt administration up-regulated the protein expression levels of these cytokines. Considering these data, our results suggested that the regulation of serum TNF-α, IL-6, and IL-1β was correlated with the expression of these cytokines in spleen.

To further investigate how the preemptive administration of Dex modulated inflammation, we measured the effects of Dex and α-Bgt on the levels of TNF-α, IL-6, and IL-1β mRNA in spleen. RT-PCR analysis revealed that LPS administration elevated the gene production of these cytokines in spleen and that pretreatment with Dex inhibited splenic TNF-α, IL-6, and IL-1β mRNA expression. However, pretreatment with α-Bgt attenuated the anti-inflammatory benefits of Dex, resulting in elevated splenic TNF-α, IL-6, and IL-1β mRNA release.

Most inflammatory signals are known to merge in the activation of the NF-κB pathway, and NF-κB has been shown to play a critical role in modulating mortality in experimental and clinical sepsis (Böhrer et al. 1997). We next determined whether the upstream NF-κB signal transduction pathway was also involved. NF-κB is a protein transcription factor and typically a heterodimer of p50 and p65 subunits. It plays a pivotal role in immune and inflammatory responses by regulating the expression of several proteins, including pro-inflammatory cytokines, chemokines, and adhesion molecules. Uncontrolled activation of the NF-κB pathway is involved in the pathogenesis of many acute and chronic inflammatory diseases. In its inactive state, the NF-κB dimer is present in the cytosol, where it is bound to an inhibitory protein, IκB. Several stimuli could induce the release and degradation of the inhibitory protein IκB from the dimeric complex (Baeuerle and Baltimore 1988), triggering a series of signaling events that result in NF-κB activation (Müller et al. 1993). Subsequently, multiple molecules involved in inflammatory responses, including TNF-α, IL-6, and IL-1β, are synthesized. Our research showed that LPS treatment enhanced IκB phosphorylation and NF-κB p65 activation in spleen and that Dex treatment hampered it. Further analysis using the selective α7nAChR antagonist α-Bgt indicated that α7nAChR activation was responsible for the decrease in IκB phosphorylation and NF-κB p65 activation. This finding corresponded with a previous report showing a similar blunting of the NF-κB pathway on the activation of the cholinergic anti-inflammatory pathway.

To our knowledge, this report provided evidence for the first time that Dex inhibited NF-κB p65 activation and lowered the TNF-α, IL-6, and IL-1β expression in levels of protein and mRNA in spleen. Conversely, pretreatment with α-Bgt reversed the anti-inflammatory capacity of Dex. According to the obtained results, we concluded that the Dex-mediated systemic inflammatory response could be due at least partly to the activation of the cholinergic anti-inflammatory pathway, resulting in decreased NF-κB activation and inflammatory cytokine production in spleen, thereby reducing the serum concentrations of inflammatory cytokines and attenuating the inflammatory responses of septic mice.

## Conclusion

Our findings demonstrated the important role of spleen in the protective effects of Dex against sepsis and provided further insight into the anti-inflammatory mechanisms of Dex. Preemptive administration of Dex was sufficiently potent to reduce inflammatory responses in LPS-induced sepsis, and future research should focus on finding clinical evidence to support the use of Dex as an anti-inflammatory adjuvant in humans.
